# Mating pattern and pollen dispersal in an advanced generation seed orchard of *Cunninghamia lanceolata* (Lamb.) Hook

**DOI:** 10.3389/fpls.2022.1042290

**Published:** 2022-10-27

**Authors:** Hanbin Wu, Shirong Zhao, Xihan Wang, Aiguo Duan, Jianguo Zhang

**Affiliations:** ^1^ State Key Laboratory of Tree Genetics and Breeding & Key Laboratory of Tree Breeding and Cultivation, National Forestry and Grassland Administration, Research Institute of Forestry, Chinese Academy of Forestry, Beijing, China; ^2^ State-owned Forestry Farm of Weimin, Shaowu, China; ^3^ Collaborative Innovation Center of Sustainable Forestry in Southern China, Nanjing Forestry University, Nanjing, China

**Keywords:** Chinese fir, genetic diversity, mating pattern, pollen contamination, seed orchard

## Abstract

Seed orchards represent the link between forest breeding and conifer production forests, and their mating patterns determine the genetic quality of seed orchard crops to a large extent. We genotyped the parental clones and their open pollination offspring in the third-generation seed orchard of Chinese fir using microsatellite markers and observed the synchronization of florescence in the seed orchard to understand the genetic diversity and mating structure of the seed orchard population. Genetic coancestry among parental clones was detected in the third generation seed orchard of Chinese fir, and the genetic diversity of the open-pollinated offspring was slightly higher than that of the parental clones. The external pollen contamination rate ranged from 10.1% to 33.7%, 80% of the offspring were produced by 44% of the parental clones in the orchard, and no evidence of selfing was found. We found that 68.1% of the effective pollination occurred within 50 m, and 19.9% of the effective pollination occurred in the nearest neighbors. We also found that successful mating requires about 30% of florescence overlap between males and females, and there was a significant positive correlation between male reproductive energy and male parental contribution. Our results provide a valuable reference for the management and design of advanced generation seed orchards.

## 1 Introduction

The tree selection breeding program aims to improve the economic value of future forests by planting excellent tree species with the highest genetic gain and diversity (El-Kassaby, 1995). A forest seed orchard is a special artificial forest composed of excellent clones or families selected manually, built according to the design requirements, and intensively managed (Hodge and White, 1993). A seed orchard is one of the most effective ways for conifer breeding; not only can it provide a large number of high-quality seeds, but it can also serve as a breeding base for improved varieties. Hence, a seed orchard is an important link in the breeding system from low to high levels (Sheng, 2014). They represent vectors that connect breeding and afforestation activities through packaging gain and the diversity of genetically improved seed crops (El-Kassaby, 1992; El-Kassaby, 2000a; El-Kassaby, 2000b). To fulfill this role, seed orchards are expected to function as closed, perfect panmictic populations (Eriksson et al., 1973), an ideal scenario that is rarely met due to the commonly observed variations in reproductive success and phenology among parental clones (Chen et al., 2019; Bian et al., 2020) as well as external gene flow (pollen contamination) from the ambient environment (El-Kassaby and Ritland, 1986; El-Kassaby, 1995). Therefore, maintaining genetic diversity in forest breeding programs is an arduous task, reflecting the trade-off between genetic diversity and genetic gain (El-Kassaby, 1995).

The mating pattern and genetic diversity level of seed orchards largely determine their adaptability, biological and abiotic resistance, and sustainability (Grattapaglia et al., 2014; Chen et al., 2018). The mating pattern of a seed orchard is mainly caused by the variation of female and male fertility between parents (Nielsen and Hansen, 2011), the difference in reproductive success rate (Torimaru et al., 2012; Funda et al., 2016), the synchronization of the flowering period (Li et al., 2011; Zhang et al., 2016; Muñoz-Gutiérrez et al., 2020), and the difference in pollen competitiveness (Nikkanen et al., 2000; Aronen et al., 2002). However, mating patterns have become more complex due to their expected co-ancestry accumulation in advanced generation breeding programs and seed orchards (Yang et al., 2021). An uneven parental gametic contribution (Chen et al., 2018), inbreeding, and pollen contamination (Wei et al., 2015; Yang et al., 2017; Song et al., 2018) may affect the genetic diversity of offspring and potential seed yield and quality.

Chinese fir (*Cunninghamia lanceolata* (Lamb.) Hook) has been deforested and utilized for about 8000 years and cultivated for more than 2000 years in China (Sheng, 2014). According to the ninth inventory of China’s forest resources, the area of Chinese fir plantation reached 9.87 million ha, and the volume reached 755 million m^3^. Genetic improvement of Chinese fir began in the 1950s. The first generation seed orchard, the second generation seed orchard, and the third generation seed orchard have been established successively, and the multi-generation genetic improvement procedure (Shi, 1994) and technical regulations for the seed orchard construction of Chinese fir (Xu et al., 2013) have been formulated. The fourth generation breeding process has started. However, with the rapid development of Chinese fir seed orchard generation, studies on parental mating patterns and flowering synchronization in the seed orchard are lacking. In addition, due to the increasing proportion of improved seeds used in afforestation activities, knowledge of the genetic variability of the seed orchard is crucial. Therefore, the evaluation of the mating mode and flowering synchronization of the seed orchard has important guiding significance for evaluating the function of seed orchards, implementing scientific management, and improving the seed yield and quality of seed orchards.

Here, we conducted a genetic analysis of parental and offspring genotypes in the third-generation seed orchard of Chinese fir in Fujian Province, China. We unraveled the population’s mating system and flowering phenology using SSR genetic markers. Our research results describe the genetic diversity and pedigree structure and depict the mating system and its influencing factors in advanced generation seed orchards. In addition, our research provides insights for future breeding plans.

## 2 Materials and methods

### 2.1 Site data

The experimental site is located in the third-generation seed orchard of Chinese fir at Weimin state-owned Forest Farm, Nanping City, Fujian Province, China (long. 117.68471°E, lat. 27.049212°N). The mean annual temperature is 18.3 °C, and the annual precipitation is 1882 mm. The seed orchard was established in 2010, covering an area of 6.4 ha. The seed orchard has 69 parents, with 1793 ramets. It consists of seven blocks with 5 m × 5 m spacing. The 69 parents were divided into two groups, A and B (i.e., 1–35 and 36–69), which were staggered in seven blocks. Each block was distributed with an adjusted random block design. The spacing between plants of the same clone was greater than 20 m, avoiding the adjacent distribution of ramets of the same clone. A pollen isolation zone (*Pinus massoniana* Lamb., *Phyllostachys edulis* (Carriere) J. Houzeau) greater than 250 m was set around the seed orchard. In November 2019, seeds were collected in blocks 3 and 4. The cones of 45 parent clones were collected (24 parent clones had no cones), and the location of the mother trees was marked. Seedlings were raised in containers at Wugong Mountain Forest Farm, Jiangxi Province, China (long. 114.247439°E, lat. 27.297688°N). In September 2021, young leaves of seedlings were collected for DNA extraction, and 2–16 individuals were collected from each family. A total of 20 family samples were collected, and 288 offspring individuals’ buds were collected (**Table S1**).

### 2.2 Genotyping

The total DNA of seed orchard parents and offspring seedlings was extracted with a CTAB plant genomic DNA rapid extraction kit (Adlai, Beijing, China). The DNA quality and concentration were detected by NanoDrop 2000. Six dinucleotides and six trinucleotide SSR markers (Li et al., 2015; Wen et al., 2015) were used for genotyping (**Table 1**). The forward primer of each primer pair was labeled with one of two fluorescent dyes (i.e., FAM or HEX) (Sangon Biotech, Shanghai, China). Polymerase chain reaction (PCR) analysis was carried out in 15 µL reaction volume: 50 ng genomic DNA template, 0.6 µL 10uM F-primer (including fluorescent primer), 0.6 µL 10uM R-primer, 12.8 µL 1.1 × Golden Star T6 Super PCR Mix (TsingKe, Beijing, China). The cycling parameters are also referred to in (Wen et al., 2015) The PCR cycling conditions consisted of an initial denaturation step of 95°C for 5 min; followed by 35 cycles of denaturation at 94°C for 30 s, annealing for 30 s (depending on the annealing temperature of the primer used, see **Table 1**), extension at 72°C for 30 s; and final extension at 72°C for 10 min. The amplification products were separated on the ABI3730 DNA analyzer (Applied Biosystems) using GeneScan-500 (LIZ) (Applied Biosystems) as an internal size standard. Allele binning and genotyping were performed automatically with Genemapper 4.0 software (Applied Biosystems) and later manually checked.

**Table 1:** Primer information and characteristics of 12 microsatellite markers were evaluated using all samples in the present study.

Locus	Primer Sequences (5’ - 3’)	Accession NO. (GenBank)	Repeat Motif	Anneal temperature (°C)	Range (bp)	Na	Ae	Ho	He	HW^1^	Reference
wx1	ATTATCCGAGGCAGATACGCAC	AB749572	(GGA)_10_	56	340-361	7	2.34	0.56	0.57	NS	Wen et al., 2015
	CTTCTCCGTATTTGATCCATCGC	AB749573									
wx2	GAGCCGTGAAGAACGAAGGTCTC	AB749574	(GAA)_12_	56	261-285	8	4.14	0.77	0.76	NS	Wen et al., 2015
	ACGATCGGATTGTCTCAGAAACG	AB749575									
wx2-3	GATCCTCTGGTACTTGGTGCCC	AB749556	(AT)_9_	56	184-196	6	1.62	0.33	0.38	NS	Wen et al., 2015
	TGCAAAGTCATGTCATCTCTGGC	AB749557									
wx2-6	TGAATGGACTGCCACAAATTCC	AB749550	(AG)_11_	56	287-311	13	3.36	0.66	0.70	NS	Wen et al., 2015
	TTCTTTGCAGGAAAGCCAACAAG	AB749551									
wx2-4	GGCTCGAGTTTGCATCTCACAC	AB749558	(TC)_9_	56	230-238	5	3.07	0.68	0.67	NS	Wen et al., 2015
	CACATCCAATCCATACAGGAGGG	AB749559									
wx4	AATGCGACTTGCAAATTTCTGG	AB749582	(AGA)_10_	56	241-262	7	1.61	0.39	0.38	NS	Wen et al., 2015
	CGAATTCCTCAATCACTTGGCTG	AB749583									
wx2-11	TGATCTTGGCATGTCAGTCTGG	AB749576	(AT)_9_	56	129-137	5	2.47	0.58	0.60	NS	Wen et al., 2015
	TGTCTGTCTGCCTGCAGTTATGC	AB749577									
wx8	TCCAGGAGTCTGTGAATCCGAAG	AB749600	(CTG)_9_	56	203-233	8	2.05	0.50	0.51	NS	Wen et al., 2015
	CAGTACCAATTCAACCCAGCAGC	AB749601									
wx2-8	CTTAAGATAGCAGCGGGAATGG	AB749562	(CT)_11_	56	240-260	11	3.24	0.49	0.69	***	Wen et al., 2015
	CTTGCTCGATTTCTTGCATCTGG	AB749563									
wx7	TTTGGGACCTTATGGAGGTGGAG	AB749602	(GGA)_9_	56	122-146	7	2.21	0.52	0.55	NS	Wen et al., 2015
	AAACCACCAGGTTGAGAAGCAGC	AB749603									
wx6	GGAGCCCTTAGAGTTACGGAG	AB749578	(ATA)_9_	56	211-223	5	2.22	0.48	0.55	NS	Wen et al., 2015
	TGGGCTCCATTCATTTGTACTGC	AB749579									
SM13	TCGTGAGTTTCTTGGTCATTTCG	KF873004	(AG)_8_	61	385-399	7	2.62	0.35	0.62	***	Li et al., 2015
	CATAAGGGTTTTCCCCACGTATA										

^1^NS, not significant; ***, significant at the 0.1% level. Na, observed number of alleles; Ae, effective number of alleles; Ho, observed heterozygosity; He, expected heterozygosity; HW, Hardy–Weinberg equilibrium.

### 2.3 Data analyses

#### 2.3.1 Genetic diversity analyses

GenALEx 6.5.1 (Peakall and Smouse, 2012) was used to calculate the genetic diversity of parents and offspring by analyzing the following parameters: observed (Na) and effective (Ae) number of alleles per locus, observed (Ho) and expected (He) heterozygosity, the Shannon diversity index (I), and fixation index (F) with F = (He – Ho)/He. The CERVUS 3.0 software (Kalinowski et al., 2007) was used to calculate parameters of genetic diversity: polymorphic information content (PIC), frequency of null alleles (Null), and Hardy–Weinberg equilibrium (HW).

#### 2.3.2 Paternity analysis

Paternity analysis was conducted using the CERVUS 3.0 software (Kalinowski et al., 2007). Ten thousand simulations were performed with 95% (strict) and 80% (loose) confidence levels. The critical LOD score was obtained by analyzing the simulated materials. The candidate father ratio and mistyped genotyping were set to 0.85 and 0.01, respectively, and the minimum number of loci was seven. The principle of CERVUS paternity analysis is that if the difference between the most probable and the second-most probable paternity exceeds a specific threshold (estimated through the simulation stage), the parent is set as candidate paternity (Kalinowski et al., 2007). We estimate the pollen contamination based on the individuals that do not match the father in the paternal analysis. The lower limit of the pollen contamination was based on the specific allele (29/288) in offspring, and the upper limit was that all unmatched individuals are produced by the peripheral pollen (97/288).

We estimated the male effective population size (Ne) following Funda et al. (2008). In order to compare the effective population size between orchards’ crops and offspring population, we also estimated Ne with the linkage disequilibrium (LD) method in the program LDNE (Waples and Do, 2008).

#### 2.3.3 Florescence

The fixed plant observation method of Bian et al. (2020) was used to observe the flower amount and flowering period. Four standard ramets with medium growth were selected for each clone in the seed orchard. The sunny side of each crown was divided into upper, middle, and lower layers. A branch with a medium flower amount was selected in each layer. The development process of male and female flowers was observed in spring, once a day until the end of the flowering period. The number of male and female flowers of the whole plant was observed at the last flowering stage. The evaluation criteria for each period of flowering of a single cone are referenced by Chen et al. (2019) and Bian et al. (2020).

The synchronization index of florescence proposed by Askew and Blush (1990) was used to evaluate the overlap of florescence. The basic idea was to evaluate the overlapping degree of the receptive and pollination stages among different genotypes. The calculation formula is given in Eq. 1:


(1) $$\;S_{ij}=\sum_{k=1}^{n}\frac{\min\left(M_{ki}\;,\;P_{kj}\right)}{\sum_{k=1}^{n}\max\left(M_{ki}\;,\;P_{kj}\right)}\;,\;\;$$

where *S_ij_* is the synchronization index of florescence of clone *i* for the male parent and clone *j* for the female parent, *M_ki_* is the loose pollen ratio of male strobilus of the *i*th grafted clone on the *k*th day; *P_kj_* is the ratio at which the *j*th grafted clone is in the fertile period on the *k*th day; *n* is the number of days from the earliest flowering to the latest ending of male and female strobilus. When the florescence of male and female parents completely overlapped, *S_ij_* = 1; when there is no overlap at all, *S_ij_* = 0; In case of partial overlap, then 0 <*S_ij_* < 1. It is worth noting that Chinese fir is a monoecious species, and the same clone can be used as both male and female parents,*S*_*ij*_ ≠  *S*_*ji*_

#### 2.3.4 Co-ancestry

Co-ancestry between parental clones was estimated using the triadic likelihood estimator (TrioML) with the software COANCESTRY (Wang, 2011). TrioML is expected to produce T of zero for unrelated cultivars, ~0.25 for half-sibs, and ~0.5 for full-sibs (Wang, 2011). Using a relatively small number of loci and possible genotyping errors reduced our estimated reliable T, so we applied TrioML to six superior cultivars of Chinese fir of known parentage (six hybrid combinations). From these cultivars, the lowest T estimated from first-degree relatives (full siblings or parent-offspring) was 0.4511; we thus used T ≥ 0.4511 as a cutoff to identify first-degree relatives. Similarly, the maximum T-value of seven parental clones from different sources selected in the three first-generation seed orchards was 0.1641. We believe that the combination with T< 0.1641 was not related. Although these values may represent the true pedigree relationship, it is also possible that other complex mating schemes, such as backcross between generations, produce T-values equivalent to first-degree relatives. The relatedness between parental clones was represented by a weightless and directionless network, plotted with the R package ‘igraph’ (Csardi and Nepusz, 2006).

## 3 Results

### 3.1 Genetic diversity of parental and offspring population

In the analysis of 12 pairs of primers, the observed number of alleles (Na) varied from 4 (wx2-4) to 9 (wx2-6) in third-generation seed orchard parental clones, with a mean value of 6.083 (**Table S2**). The mean effective number of alleles (Ae) was 2.615. Among loci, observed heterozygosity (Ho) and expected heterozygosity (He) ranged from 0.375 to 0.853 and from 0.397 to 0.793, with means of 0.528 and 0.572, respectively. Primer wx2-8 showed significant deviation from Hardy–Weinberg equilibrium. A total of 80 alleles were amplified from 12 microsatellite loci in 288 Chinese fir offspring, with an average of 6.667 per primer (**Table S3**). Ho and He values ranged from 0.439 to 0.835 and 0.412 to 0.741, with means of 0.614 and 0.574, respectively. Loci wx1, wx2, wx2-8, wx2-4 and SM13 significantly deviated from the Hardy–Weinberg equilibrium. Compared to the parental population, the observed number of alleles (Na), and the proportion of observed heterozygosity in the offspring displayed an increase(Ho=0.528 and He=0.572, Ho=0.614 and He=0.574).r

### 3.2 Co-ancestry between parental clones

**Figure 1:**
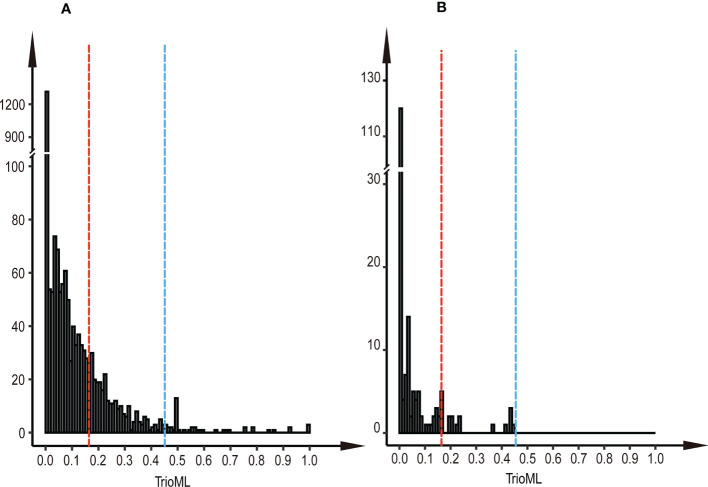
Histogram of paired TrioML values. **(A)** Histogram of paired TrioML values of parental clones in the third-generation seed orchard. **(B)** Histogram of paired TrioML values between parents with 191 cases of mating success. The red dotted line represents the unrelated cutoff of the parental clones (T = 0.1641), and the blue dotted line represents the threshold above which pairwise comparisons were considered first-degree relatives (T = 0.4511).

**Figure 2:**
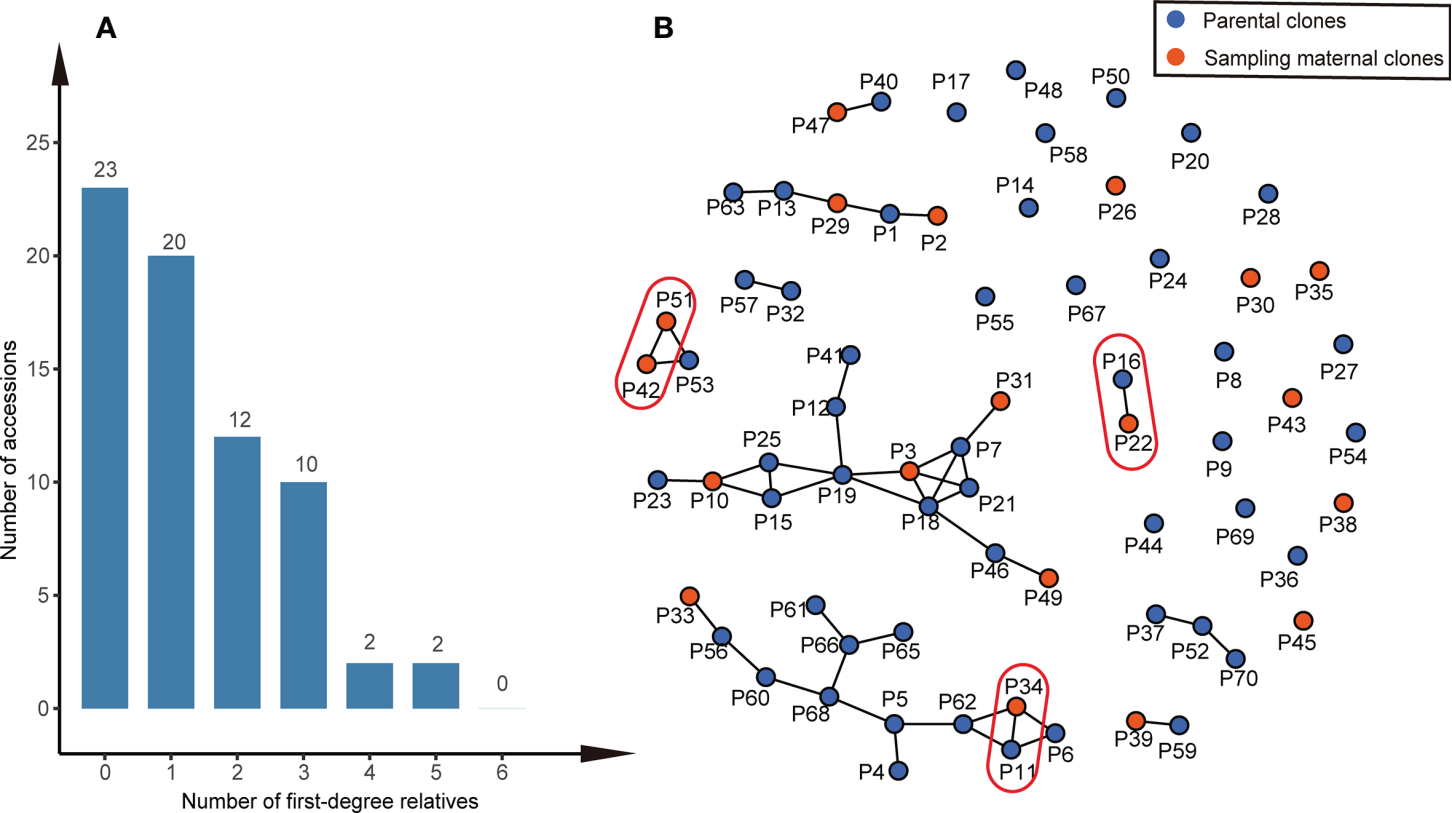
First-degree relationships within the Chinese fir germplasm collection. **(A)** Histogram of the number of first-degree relatives in the thirdgeneration seed orchard of Chinese fir. **(B)** A network of parental clones shows connections between first-degree relatives. The T (TrioML) of the parental clones in the red oval was one, and they were considered clones of one another.

**Figure 3:**
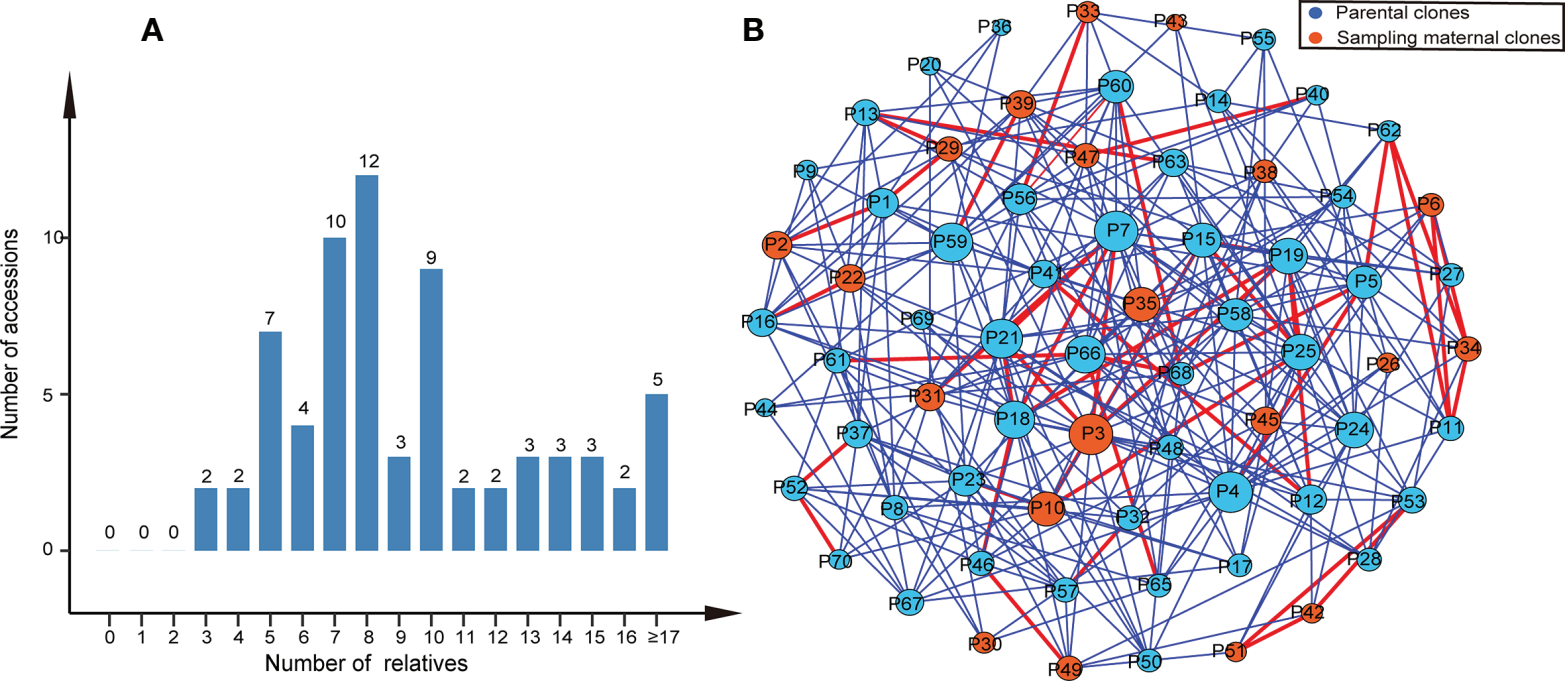
Relatives within the Chinese fir germplasm collection. **(A)** Histogram of the number of relatives in the third-generation seed orchard of Chinese fir. **(B)** A network of parental clones showing connections between relatives. The two circles connected by the red lines represent parental clone pairs were first-degree relatives, and the blue lines represent that parental clone pairs were related but probably not first-degree relatives. The larger the circle, the more parental clones were related to it.

We found that most parental clone pairs were uncorrelated (86.19%), and the estimated T was less than 0.1641 (**Figure 1A**). In fact, most of the combinations estimated T = 0 (56.01%) or less than 0.1 (77.15%). In addition, 11.85% of the combinations were between 0.1641 and 0.4511 (**Figure 1A**), indicating that they are related but may not be first-degree relatives and may be half-sib. Finally, 1.96% of the combinations were identified as first-degree relatives, of which the estimated T of the three combinations was equal to one (**Figure 2B**), so we were led to believe that they are clones of each other. Overall, 46 (65.7%) of the third-generation seed orchard parental clones had at least one first-degree relative (**Figure 2A**); P18 and P19 had five close relatives, followed by P3 and P7, which had four first-degree relatives. Any parental clone of the seed orchard had at least three related parental clones in the seed orchard (**Figure 3A**); P3, P4, and P7 had 18 parental clones related to them (**Figure 3B**).

### 3.3 Paternal analysis of the open-pollinated offspring

**Figure 4:**
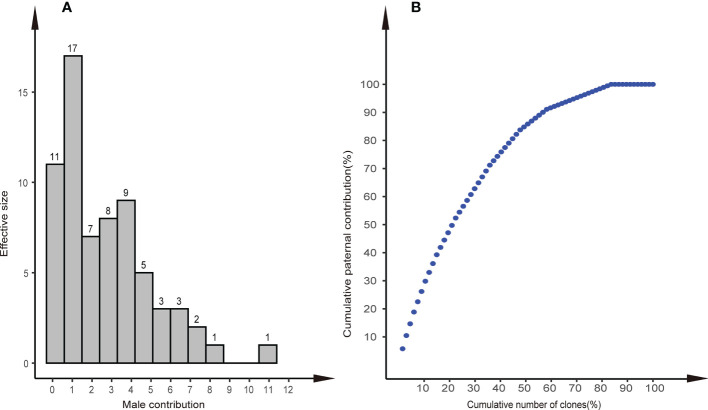
Distribution of paternal contributions and cumulative paternal contribution. **(A)** Distribution of paternal contributions of the 58 identified paternal clones in the Chinese fir clonal seed orchard. **(B)** Relationship between the cumulative number of clones (%) and cumulative paternal contribution (%) in the seed orchard.

The results of parental analysis of 288 offspring showed that no selfing was found, and the outcrossing rate was 100% (**Table 2**). There were 191 (66.3%) offspring that were matched to the paternal clones, the remaining 97 offspring individuals were not matched, and 29 individuals in the offspring population found alleles (Na) unique to the offspring that did not appear in the parental population, which indicated that the pollen contamination rate of the seed orchard was 10.1%–33.7%, the average pollen contamination of the family was 32.3% ± 3.6%. There were 58 (86.6%) parental clones that participated in pollination. The effective pollination times of clones ranged from 1 to 11 (**Figure 4A**). The parental clone called P49 had the most pollination times, with a contribution rate of 5.76%. We found that 44% of the paternal clones produced 80% of the offspring, which indicated that the contribution rate of paternal clones in the seed orchard was unbalanced (**Figure 4B**).

**Table 2:** Paternity analysis for the 288 offspring.

Maternal clone	Number of offspring	Number of assigned offspring	Rate (%)	Number of assigned paternal clones	Assigned paternal diversity (%)	Outcrossing rate (%)
P10	15	10	66.67	5	50.00	100
P2	15	8	53.33	8	100.00	100
P22	15	14	93.33	12	85.71	100
P26	16	12	75.00	9	75.00	100
P29	15	7	46.67	6	85.71	100
P3	15	6	40.00	6	100.00	100
P30	15	13	86.67	9	69.23	100
P31	15	9	60.00	8	88.89	100
P33	15	9	60.00	5	55.56	100
P34	15	8	53.33	7	87.50	100
P35	15	11	73.33	8	72.73	100
P38	15	10	66.67	9	90.00	100
P39	15	11	73.33	7	63.64	100
P42	15	14	93.33	12	85.71	100
P43	15	11	73.33	11	100.00	100
P45	15	9	60.00	7	77.78	100
P47	15	9	66.67	7	77.78	100
P49	15	8	53.33	6	75.00	100
P51	15	10	66.67	8	80.00	100
P6	2	2	100.00	2	100.00	100
Total	288	191	—	152	—	—

Male effective population of seed orchard offspring (♂Ne) was 37.57, and ♂Ne/N ratio was 0.54 (**Table S4**). If it is assumed that each pollen contamination involved a single father, the ♂Ne increased considerably (by 103%). The Ne of parents and offspring population in seed orchard estimated by LD method was 49.00 and 62.30 respectively.

### 3.4 Effective pollen transmission distance

The effective propagation of pollen among clones in the seed orchard was random, and there was no obvious specific direction for pollination. The distance between each mother tree and its corresponding pollen donor was 5–176 m, and the average propagation distance was 49 m (**Table 3**); 68.1% of the effective pollination was within 50 m, and 19.9% of the effective pollination occurred in adjacent areas. All maternal clones showed substantial variation in mean pollination distance, with a mean coefficient of variation of pollination distance of 84.6% (**Table 3**).

**Table 3:** The difference in mean pollen transmission distance among female parent clones.

Maternal clone	Number of assigned paternal clones	Mean (m)	St. Error	CV (%)
P10	10	36.69	5.11	13.94
P2	8	42.62	49.23	115.51
P22	14	58.58	46.90	86.21
P26	12	32.44	30.71	104.98
P29	7	42.02	38.58	89.65
P3	6	72.99	72.78	99.70
P30	13	35.64	41.11	120.30
P31	9	36.44	36.43	100.21
P33	9	63.52	34.52	54.34
P34	8	79.14	42.51	60.86
P35	11	50.67	48.12	89.04
P38	10	55.89	44.63	90.22
P39	11	33.99	27.87	82.00
P42	14	40.33	41.18	102.11
P43	11	61.47	18.99	38.85
P45	9	22.80	36.22	158.86
P47	9	40.79	35.65	49.93
P49	8	53.14	46.18	85.67
P51	10	74.38	50.40	74.82
P6	2	95.49	28.97	30.34
Total	191	46.91	40.79	84.60

### 3.5 Florescence synchronization

**Figure 5:**
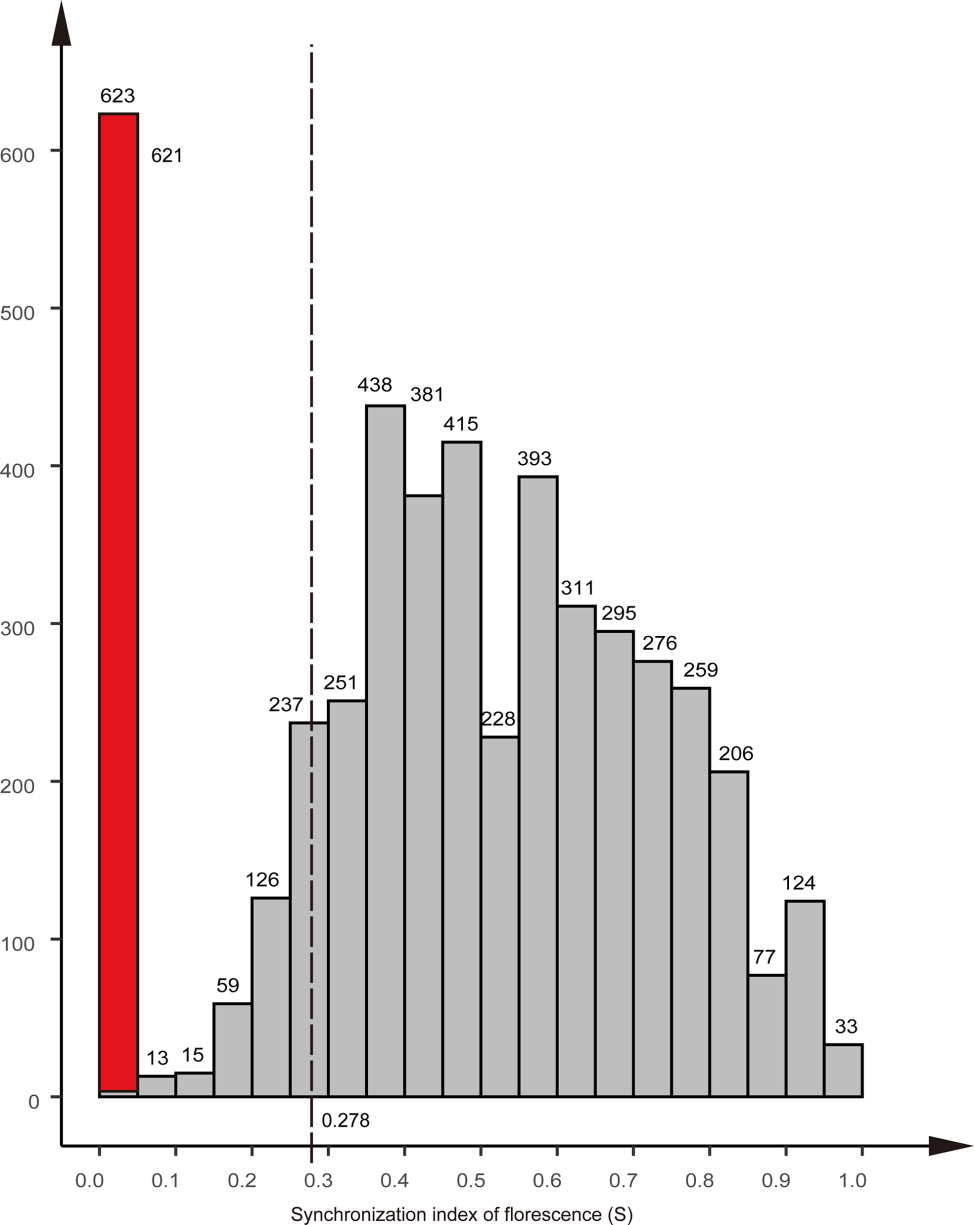
Distribution of the synchronization index of florescence (S) of the clones in the Chinese r clonal seed orchard. The red column indicates the nine clones that did not produce female flowers. equal to zero; The dotted line indicates the lowest S-value of mating success.

The synchronization index of florescence (*S*) for the Chinese fir parental clones was 0.48, with a minimum value of 0.045 and a maximum value of 1.00. Nine clones did not produce female flowers; consequently, their *S*-values were set to zero (red column in **Figure 5**). In the 191 cases of successful reproduction, the lowest *S*-value was 0.278 (dotted line in **Figure 5**). Excluding the clonal parents that did not produce female flowers, only 7% of the mating combinations in the seed orchard had an *S*-value lower than 0.278.

### 3.6 Mating success

**Figure 6:**
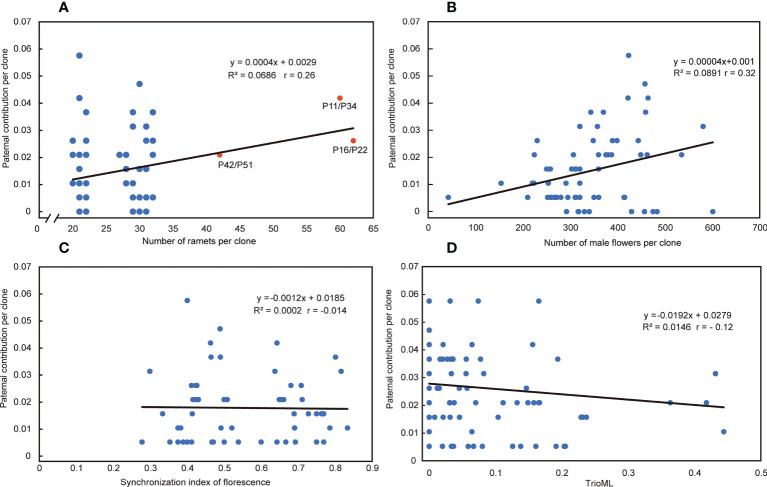
Relationship between reproductive factors and paternal contribution. **(A)** Relationship between the number of ramets per clone and paternal contribution per clone in the seed orchard. Red dots represent that pairwise comparisons were considered clones. **(B)** Relationship between the number of male flowers per clone and paternal contribution per clone in the seed orchard. **(C)** Relationship between the synchronization index of florescence (S) and the paternal contribution per clone in the seed orchard. **(D)** Relationship between the T (TrioML) and the paternal contribution per clone in the seed orchard. R2= R-Squared; r= Pearson correlation coefficient.

The paternal contribution of clones was significantly different (Chi-square test, p<0.001), and the number of ramets of clones explained 6.86% of the paternal contribution (**Figure 6A**). There was a significant difference in the number of flowers per clone among clones in the seed orchard (ANOVA, p<0.001; **Table S5**), and a positive correlation between the number of male flowers per clone and their paternal contribution rate (r = 0.32, p = 0.0141); their pollen yield explained 8.91% of the paternal contribution (**Figure 6B**). The *S*-value of mating success was not related to its paternal contribution (**Figure 6C**). There was a weak negative correlation between paternal contribution and T value of parents with mating success (r = - 0.12, p = 0.082) (**Figure 6D**). In addition, we found that the T-value of most mating parents (91.7%) was less than 0.1641 in 191 cases of mating success, which indicated that most parental clones of mating success were not related, and 8.3% of parents were related but did not reach the level of first-degree relatives (**Figure 1B**).

## 4 Discussion

### 4.1 Genetic diversity and co-ancestry

The main function of forest seed orchards is to produce a large number of genetically improved seeds without reducing genetic diversity (Chaloupková et al., 2019). For a long rotation species, the area where the species is planted should monitor the deployment of improved Germplasm to ensure that the level of genetic diversity is maintained and equivalent to the natural regeneration stand (Ruņģis et al., 2019). In this study, we compared the genetic diversity indexes of parental clones and offspring populations in the third-generation seed orchard of Chinese fir trees. The results show that the genetic diversity of parental clones and offspring was high, the mean observed heterozygosity (Ho) and expected heterozygosity (He) of parental clones were 0.528 and 0.572, and other coniferous seed orchard plants also had similar levels (*Larix kaempferi*, He = 0.525; *Larix olgensis*, He = 0.5833) (Wei et al., 2015; Chen et al., 2018). The expected heterozygosity (He) was slightly lower than that of the Chinese fir provenance population in the same province (He = 0.625) (Duan et al., 2017), which may be related to the population size and the generations of Chinese fir seed orchard. One study showed that the genetic diversity of the advanced generation breeding population gradually decreased as breeding progressed (Li et al., 2020). The Ho and He of the free pollinated offspring in the Chinese fir third-generation seed orchard were 0.614 and 0.575, respectively. The genetic diversity of the free-pollinated offspring was slightly higher than that of the parental clones. Similar reports have also been made on *Picea abies* (L.) Karst. (Sønstebø et al., 2018), *L. kaempferi* (Chen et al., 2018), and other tree species (Chaix et al., 2003; Yang et al., 2016). The genetic diversity of the progenies was higher than that of the parental clones, which was related to the introduction of new alleles and haplotypes by external pollen (Kess and El-Kassaby, 2015). In this study, the seed orchard had high pollen pollution (10.1%–33.7%).

Mating between relatives in the seed orchard population usually leads to the reduction of seed yield and the performance of inbreeding seedlings, which will continue throughout the tree life cycle (Woods and Heaman, 1989; Woods et al., 2001; Wang et al., 2004; Stoehr et al., 2014). In this study, genetic coancestry among parental clones was detected in the third generation seed orchard of Chinese fir, more than half (65.71%) of the parental clones had at least one first-degree relative, and any parental clone of the seed orchard had at least three related parental clones in the seed orchard. The build-up of co-ancestry had also been found in the advanced generation seed orchards of *Pinus tabuliformis* Carrière and *Pinus sylvestris* var. mongolica (Yang et al., 2020; Yang et al., 2021). This phenomenon of the build-up of co-ancestry was related to the determination of the progeny in the seed orchards and the selection of breeding populations. The families with the best performance often contributed more selected individuals than those with poor performance (Yang et al., 2021). The understanding of the parental relationship in seed orchards not only has important guiding significance for the breeding plan and process but also can be used as the basis for the spatial deployment design of parental clones in seed orchards.

### 4.2 Selfing and pollen contamination

Most trees are outbreeding species that have developed different mechanisms against selfing: dioecism, gametophytic self-incompatibility, the avoidance of contemporary flowering of male and female parts in hermaphrodite flowers, the separation of female and male flowers in different parts of the crown, or frequent abortion of embryos after selfing resulting in empty seeds (Liesebach et al., 2021). Selfing will cause recession, resulting in higher seedling mortality, etiolated seedlings, and poor individual vitality (Moriguchi et al., 2005). Therefore, evaluating and reducing the selfing rate of seed orchards in such ideal closed environments is very important.

It has been reported that the selfing rate of most conifer seed orchards is at low levels, with 5.1% for *Pinus sylvestris* L. (Funda et al., 2015), 0.45% for *Platycladus orientalis* (Huang et al., 2018), 1.85% for *L. olgensis* (Wei et al., 2015), 1.74%–6% for *P. abies* (L.) Karst. (Sønstebø et al., 2018); but there are exceptions, the selfing rate of a *Pseudotsuga menziesii* (Mirb.) Franco seed orchard is 12%–17% (Korecký and El-Kassaby, 2016; Song et al., 2018). Selfing was not detected in this study. On the one hand, it was related to the configuration of the seed orchard, and the distance between ramets of the same clone was more than 20 m; on the other hand, it is related to the tree species characteristics of Chinese fir (wind pollination), as female flowers in the phenological period mostly grow at the top of the trees (Zhang, 2005), and the seed orchard has auxiliary pollination measures. Similarly, selfing was not detected in the 1.5th generation seed orchard of red-heart Chinese fir (Chen et al., 2021). Previous studies have found that the self-pollination of Chinese fir can produce normally developed offspring (Xu et al., 2015; He et al., 2016), but all the cases of self-pollination were controlled pollination (only the pollen of its own clones). We speculated that the mating of open pollination of Chinese fir might be related to the affinity of pollen, as female flowers may preferentially choose the pollen of unrelated clones in the fertilization process. When the pollen of other clones is low or absent, plant-selective selfing provides reproductive security for the population. However, this conjecture about Chinese fir needs to be verified by further research. We also found low levels of inbreeding (**Figure 6B**). Inbreeding has generally been regarded as something to avoid in seed orchards, lesser relatedness between parents would lead to more successful fruiting, with inbreeding depression expressed only later in the established plantation (Mullin et al., 2019). It is generally recommended to manage the breeding population with separate sublines so that individual, unrelated seed orchard parents can be selected from each subline (McKeand and Bridgwater, 1998). This way focus on the gene pool of the seed orchard crop from the orchard. In an addition, Lindgren et al. (2009) recommended that under the constraint of overall genetic diversity (or number of states), the relatedness of pairs of seed-orchard parents were penalized to reduce the expected inbreeding.

Pollen contamination from outside the seed orchard tends to increase the genetic diversity of the seed orchard with a small number of parental clones (El-Kassaby and Ritland, 1986; Lindgren and Prescher, 2005), but it may also lead to the introduction of poor genetic material, so it should be prevented (Pakull et al., 2021). The mean pollen contamination in the two seed orchards of *P. abies* (L.) Karst. was 22.9%, *P. menziesii* (Mirb.) Franco seed orchards reported pollen contamination rates of 10%–18%, and up to 28% under natural conditions (Sk Lai et al., 2010; Kess and El-Kassaby, 2015; Korecký and El-Kassaby, 2016; Song et al., 2018). In this study, even if there was an isolation zone far greater than the mean pollen transmission distance, the pollen contamination rate of the third-generation seed orchard of Chinese fir was between 10.1% and 33.7%; moreover, the unique allele (Na) of the offspring was also observed. We speculate that there was a large area of farmland between blocks 3-4 and 6-7 where the farmers planted Chinese fir on farmland after the farmland was abandoned, and these trees reached the stage of reproductive development. We believe that these Chinese firs were the source of pollen contamination donors.

Knowledge about the effective population size and pollen pollution is crucial to the establishment and management of seed orchards (Sønstebø et al., 2018). The effective population size is lower than the number of parents in the seed orchard (i.e., the number of parents), and the ratio of male effective population size to the census population size (♂Ne/N) was 0.54 in the seed orchard, which is consistent with most previous studies on the seed orchard (Funda and El-Kassaby, 2012; Sønstebø et al., 2018). The Ne estimated by LD method was larger than that estimated by paternity analysis (♂Ne), which is related to the high level of pollen contamination in the seed orchard, the pollen contamination increased the effective population size (Nikkanen and Ruotsalainen, 2000).

### 4.3 Paternal contribution and pollen transmission distance

It is generally believed that equal paternal contribution is very important for improving the genetic quality of seeds (El-Kassaby and Reynolds, 1990; Wheeler and Jech, 1992; Stoehr et al., 1998). Equal parental representativeness tends to produce the same gamete contribution as the parents in the seed orchard, thus reflecting their allele frequencies and meeting the basic quantitative genetic hypothesis of trait generation transmission (El-Kassaby and Sziklai, 1982). However, this is an ideal state based on the assumption of an equal reproductive yield of parents (male and female gametes) (Moriguchi et al., 2004). In this study, the paternal contribution rate of the third-generation Chinese fir seed orchard was in an unbalanced state, 80% of the gamete contribution comes from 44% of the parental population, and a similar proportion has been reported in other conifer seed orchards (Sk Lai et al., 2010; Song et al., 2018; Pakull et al., 2021; Heuchel et al., 2022). The reproductive success of the male parent was related to the number of ramets of the clone and the distribution of the male parent of the clone, Torimaru et al.(2012) found that the number of ramets of each clone was positively correlated with their paternal contribution in *P. sylvestris* seed orchard. In this study, we also found this positive correlation (r = 0.26), and the paternal contribution of clones was significantly different. The number of ramets of clones explained relatively little (6.86%) for the contribution of male parents. The mating success of male parents also depends on the pollen yield, flowering phenology, germination vigor of pollen grains, germination time, pollen tube growth rate, selective fertilization, and other traits (Aronen et al., 2002). Still, the major premise was that male and female florescence overlap. Our study showed that the mean *S*-value was 0.555 and the minimum *S*-value was 0.278 in 191 successful mating cases, which indicates that pollination can be completed so long as there is about a 30% overlap in florescence, even though there was no obvious correlation between the *S*-values and the paternal contribution rate (**Figure 6C**). It follows that the flowering phenology was not the main reason for the unbalanced paternal contribution. There was a significant difference in the number of male flowers among clones in the seed orchard (p<0.001) and a positive correlation between the number of male flowers per clone and their paternal contribution rate (r = 0.32). The pollen yield explained 8.91% of the contribution of male parents, noting that a higher proportion (~75%) of the more accurate index (pollen weight per clone) was used in the *P. sylvestris* L. seed orchard (Torimaru et al., 2012).

Knowledge of the effective pollination distance and mating system is important for the protection and management decisions of seed orchards and tree populations (Sork et al., 2002; Funda et al., 2008). In this study, the mean effective pollination distance of the third-generation seed orchard of Chinese fir was 47 m, and the longest distance was 176 m; 65.4% of the effective pollination occurred within 50 m, and 19.9% of the effective pollination occurred in the neighborhood, thus supporting the pollen transmission mode of short distance transmission. The pollination distance of Chinese fir maternal clones depends on their position in the seed orchard and the affinity between mating gametes. The wind direction (Feng et al., 2010), meteorological factors, (Huang et al., 2018) and management measures (such as auxiliary pollination) of the seed orchard during the flowering period will have an impact on the mattings. Pollen movement and embryo competition are complex processes. Previous studies on the pollen transmission of Chinese fir have shown that the pollen diffusion distance of Chinese fir is between 150 m and 600 m (Jiang et al., 1986; Chen, 1991; Chen et al., 1996). This gap may be due to different research methods. Previous studies mostly focused on direct observations of pollen density at different locations. The effectiveness and accuracy of this method were unstable and, in most cases, will not truly reflect the actual pollination and gene flow. The pollen dispersion law also depends on the population size, spatial distribution, and habitat characteristics (Ou, 2012).

### 4.4 Implications for the management and spatial design of a Chinese fir advanced generation seed orchard

Evaluation of mating patterns and pedigree structure of seed orchards can provide valuable information for scientific management and layout design of seed orchards. The limited number of marker loci used in this study, together with the difficulty of correctly scoring null alleles in these marker systems may have constrained assignment precision (Funda et al., 2015). SNP (single nucleotide polymorphism) analyses based on hundreds or thousands of markers have led to a stronger power of precise parentage reconstruction than microsatellite SSRs (Laucou et al., 2018). They have become more and more popular because they can be high throughput genotyping through next-generation sequencing (NGS) at a moderate cost (Zheng et al., 2019). The application of this technology in higher-generation seed orchard populations should be strengthened in future studies.

As the material for paternity analysis is the normal growth and development of the seed orchard’s progeny population, most empty and astringent seeds (inactive) or seeds containing lethal alleles were produced by selfing, and the death of seeds at the germination stage was excluded (González-Martínez et al., 2001). Therefore, the actual selfing rate may be higher than reported in this study. The size limit of the offspring population may also ignore some successful mating cases, but the entire study simulates the production process of advanced generation seed orchards. From flowering and mating to the offspring plants with normal development, it can provide effective guidance for production practice.

Based on the universality of tree species distribution, seed orchards have been established in both marginal and central production areas. The mating systems of seed orchards are different under the trend of climate in different distribution areas and global climates. Therefore, a more comprehensive study is needed in different regions and years in future studies. Due to the annual and seasonal differences in the reproductive capacity of parents in seed orchards (Funda et al., 2009; Muñoz-Gutiérrez et al., 2020), continuous observation of mating and florescence in seed orchards is greatly significant to the management and construction of seed orchards and the formulation of breeding policies. However, continuous observation is a laborious and costly study. The individual contributions of parents in *P. abies* (L.) Karst. seed orchard was well correlated between the two studied years (Sønstebø et al., 2018). In this study, the number of ramets per clone and the yield of male flowers are positively related to the paternal contribution. The arrangement of the unequal number of plants could be considered in the seed orchard configuration design when establishing the seed orchard, and the reproductive capacity can be used as a predictor of the high yield of parents in the seed orchard.

A higher pollen pollution rate will reduce the genetic gain of offspring. Additional seed orchard management measures are needed to avoid high pollution rates. A first step in avoiding pollen pollution would be to remove the single Chinese fir tree near the seed orchard, implement intensive management of the seed orchard, and set up isolation areas. Although the effective transmission distance of pollen in this study was 5–176 m, the pollen particles were small and light (Ma et al., 2017), and the dispersion distance was greater than 600 m (Chen et al., 1996), which justifies that the isolation area should be greater than 600 m. Although the mating success of Chinese fir had a loose male and female florescence overlap (about 30%), the uneven male parental contribution may lead to the seeds having common ancestors and reduce the stability and adaptability of the progeny stand. Therefore, it is necessary to implement pollen management strategies, such as controlling pollination and supplementing large-scale pollination, to improve the genetic quality of the produced seeds (Kaya et al., 2006; Stoehr et al., 2006; Fernandes et al., 2008; Funda and El-Kassaby, 2012; Chen et al., 2018). Management measures such as delaying flowering can be implemented for parents who do not encounter flowering (Funda and El-Kassaby, 2012). Inbreeding should be avoided in seed orchard production, but inbreeding is inevitable in recurrent selection. This study found that most matings occur within a given distance, and 19.90% occur in the nearest neighbors in the advanced seed orchard of Chinese fir. Moreover, we also found nearly 10% of inbreeding. Therefore, it is requisite to design the layout of seed orchards to separate related parental clones to maximize the spatial distance between them and minimize the impact of inbreeding. Traditional methods, such as adjusted random block design and grouping arrangement, were often used in the layout of Chinese fir seed orchards, but are no longer used in advanced generation seed orchards. Based on the development of computer algorithms, many excellent seed orchard layout designs have emerged, such as COOL (Bell and Fletcher, 1978), MI (Lstibůrek and El-Kassaby, 2010; Lstibůrek et al., 2015), R^2^SCR (El-Kassaby et al., 2014), ONA (Chaloupková et al., 2016), and IAPGA (Yang et al., 2020). However, the meeting of male and female gametes at florescence is the premise of mating, and the amount of male and female flowers is the embodiment of fertility. In recent years, new bionic intelligent algorithms have emerged and been optimized, making it possible to design the layout of complex advanced generation seed orchards that combine relationship, florescence, and flower amount.

## 5 Conclusion

Monitoring genetic diversity and mating patterns is especially important in high-generation seed orchards. Correlation studies of parental clones in seed orchards and pollination dynamics could prove helpful in making management decisions aimed at improving the genetic quality of seeds and in protecting the genetic resources of seed orchards. Our conclusions are as follows. (1) Genetic coancestry among parental clones was detected in the third generation seed orchard of Chinese fir; (2) The seed orchard had a high level of outcrossing (100%), and no selfing offspring were found; (3) The parental contribution in the Chinese fir seed orchard was unbalanced, and the male parental contribution showed a significant positive correlation with the number of ramets of the clone and the number of male flowers of the clone, but was not correlated with the flowering synchronization; (4) The efficient pollination of seed orchards mostly occurs at close range. In addition, we also found a small proportion of mating between related clones. Assessment of pollination dynamics should become a routine procedure in advanced generation seed orchard management to monitor the progress of orchard crop inbreeding and fitness. The results from this research are significant for the management and construction of seed orchards and the formulation of new breeding strategies in the future.

## Data availability statement

The datasets presented in this study can be found in online repositories. The names of the repository/repositories and accession number(s) can be found below in the article.

## Author contributions

AD and HW designed the experiments. HW, SZ and XW participated in data collection and phenotype measurement. AD and SZ participated in data analysis and processing. AD and JZ conceived the project and obtained fundings. JZ performed the field management. HW wrote the manuscript. All authors contributed to the article and approved the submitted version.

## Funding

This research was supported by the national key research and development project of China of the “14th five-year plan”: “Research on Breeding of high-yield, high-quality and high-efficiency new varieties of Chinese Fir”.

## Acknowledgments

The authors are grateful to the anonymous reviewers and handling Editor for their constructive comments to improve the manuscript.

## Conflict of interest

The authors declare that the research was conducted in the absence of any commercial or financial relationships that could be construed as a potential conflict of interest.

## Publisher’s note

All claims expressed in this article are solely those of the authors and do not necessarily represent those of their affiliated organizations, or those of the publisher, the editors and the reviewers. Any product that may be evaluated in this article, or claim that may be made by its manufacturer, is not guaranteed or endorsed by the publisher.
