# Supplementary material for: Mating pattern and pollen dispersal in an advanced generation seed orchard of Cunninghamia lanceolata (Lamb.) Hook
Source: Front Plant Sci. 2022 Oct 27;13:1042290. doi: 10.3389/fpls.2022.1042290 (PMC9646989; doi:10.3389/fpls.2022.1042290)
Supplement: Supplementary file 3 [file DataSheet_3.docx]

**Table S1** Summary of parental clones and offspring population of third-generation seed orchard of Chinese fir.

| Parental clones | Parent clones with cones | Cone yield per ramet (g) | Seed yield per ramet (g) | 100 grain weight (g) | Parent clones with offspring seedlings | Sample size |
| --- | --- | --- | --- | --- | --- | --- |
| P1 | Yes | 129.67 | 2.66 | 0.41 | No | — |
| P2 | Yes | 78.05 | 1.28 | 0.57 | Yes | 15 |
| P3 | Yes | 45.00 | 1.47 | 0.32 | Yes | 15 |
| P4 | No | — | — | — | — | — |
| P5 | Yes | 117.73 | 2.32 | 0.40 | No | — |
| P6 | Yes | 48.00 | 1.49 | 0.56 | Yes | 2 |
| P7 | Yes | 28.83 | 0.47 | 0.33 | No | — |
| P8 | Yes | 46.23 | 0.51 | 0.38 | No | — |
| P9 | No | — | — | — | — | — |
| P10 | Yes | 58.43 | 1.24 | 0.45 | Yes | 15 |
| P11 | Yes | 66.60 | 0.91 | 0.54 | No | — |
| P12 | No | — | — | — | — | — |
| P13 | Yes | 160.00 | 6.25 | 0.43 | No | — |
| P14 | No | — | — | — | — | — |
| P15 | No | — | — | — | — | — |
| P16 | Yes | 42.00 | 0.27 | 0.27 | No | — |
| P17 | No | — | — | — | — | — |
| P18 | No | — | — | — | — | — |
| P19 | Yes | 46.80 | 2.76 | 0.48 | No | — |
| P20 | No | — | — | — | — | — |
| P21 | No | — | — | — | — | — |
| P22 | Yes | 243.30 | 3.28 | 0.38 | Yes | 15 |
| P23 | No | — | — | — | — | — |
| P24 | No | — | — | — | — | — |
| P25 | No | — | — | — | — | — |
| P26 | Yes | 381.25 | 4.68 | 0.47 | Yes | 16 |
| P27 | Yes | 62.10 | 0.88 | 0.50 | No | — |
| P28 | Yes | 79.93 | 1.25 | 0.41 | No | — |
| P29 | Yes | 179.83 | 6.40 | 0.75 | Yes | 15 |
| P30 | Yes | 255.00 | 2.27 | 0.64 | Yes | 15 |
| P31 | Yes | 443.90 | 5.32 | 0.61 | Yes | 15 |
| P32 | No | — | — | — | — | — |
| P33 | Yes | 70.68 | 2.03 | 0.41 | Yes | 15 |
| P34 | Yes | 64.03 | 1.98 | 0.39 | Yes | 15 |
| P35 | Yes | 48.98 | 1.02 | 0.38 | Yes | 15 |
| P36 | No | — | — | — | — | — |
| P37 | Yes | 71.60 | 1.32 | 0.56 | No | — |
| P38 | Yes | 254.87 | 2.76 | 0.57 | Yes | 15 |
| *(To be continued on the next page)* | | | | | | |
| *(Continued)* | | | | | | |
| Parental clones | Parent clones with cones | Cone yield per ramet (g) | Seed yield per ramet (g) | 100 grain weight (g) | Parent clones with offspring seedlings | Sample size |
| P39 | Yes | 77.13 | 2.05 | 0.56 | Yes | 15 |
| P40 | No | — | — | — | — | — |
| P41 | No | — | — | — | — | — |
| P42 | Yes | 45.80 | 1.76 | 0.62 | Yes | 15 |
| P43 | Yes | 52.75 | 1.40 | 0.55 | Yes | 15 |
| P44 | Yes | 20.40 | 0.35 | 0.30 | No | — |
| P45 | Yes | 36.60 | 0.88 | 0.48 | Yes | 15 |
| P46 | No | — | — | — | — | — |
| P47 | Yes | 235.33 | 2.51 | 0.43 | Yes | 15 |
| P48 | No | — | — | — | — | — |
| P49 | Yes | 353.63 | 5.84 | 0.41 | Yes | 15 |
| P50 | Yes | 211.47 | 4.72 | 0.45 | No | — |
| P51 | Yes | 65.97 | 1.91 | 0.41 | Yes | 15 |
| P52 | No | — | — | — | — | — |
| P53 | Yes | 305.25 | 3.41 | 0.25 | No | — |
| P54 | No | — | — | — | — | — |
| P55 | Yes | 62.05 | 1.09 | 0.34 | No | — |
| P56 | Yes | 71.75 | 0.66 | 0.41 | No | — |
| P57 | Yes | 385.90 | 4.97 | 0.49 | No | — |
| P58 | Yes | 49.80 | 1.66 | 0.64 | No | — |
| P59 | Yes | 290.13 | 5.28 | 0.39 | No | — |
| P60 | Yes | 104.57 | 2.69 | 0.41 | No | — |
| P61 | Yes | 297.18 | 3.67 | 0.49 | No | — |
| P62 | No | — | — | — | — | — |
| P63 | Yes | 109.47 | 2.17 | 0.43 | No | — |
| P65 | Yes | 376.80 | 4.93 | 0.49 | No | — |
| P66 | No | — | — | — | — | — |
| P67 | No | — | — | — | — | — |
| P68 | No | — | — | — | — | — |
| P69 | Yes | 455.27 | 6.55 | 0.58 | No | — |
| P70 | Yes | 466.53 | 7.96 | 0.43 | No | — |

**Table S2** Diversity parameters for the Chinese fir parental clones. Na, observed number of alleles; Ae, effective number of alleles; Ho, observed heterozygosity; He, expected heterozygosity; HW, Hardy–Weinberg equilibrium, F(Null), estimate of null allele frequency. NS = not significant, * = significant at the 5% level, ** = significant at the 1% level, *** = significant at the 0.1% level.

| Locus | Na | Ae | I | Ho | He | F | HW | F(Null) |
| --- | --- | --- | --- | --- | --- | --- | --- | --- |
| wx1 | 7.00 | 2.50 | 1.20 | 0.55 | 0.60 | 0.08 | NS | 0.06 |
| wx2 | 8.00 | 4.83 | 1.72 | 0.85 | 0.79 | -0.08 | NS | -0.04 |
| wx2-3 | 5.00 | 1.71 | 0.81 | 0.39 | 0.41 | 0.05 | NS | 0.03 |
| wx2-6 | 9.00 | 3.24 | 1.45 | 0.66 | 0.69 | 0.04 | NS | 0.02 |
| wx6 | 5.00 | 1.90 | 0.87 | 0.51 | 0.47 | -0.07 | NS | -0.03 |
| wx7 | 6.00 | 1.98 | 1.00 | 0.48 | 0.49 | 0.03 | NS | 0.00 |
| wx2-11 | 5.00 | 2.39 | 1.00 | 0.59 | 0.58 | -0.01 | NS | 0.00 |
| wx2-8 | 7.00 | 4.12 | 1.57 | 0.38 | 0.76 | 0.51 | *** | 0.33 |
| wx8 | 7.00 | 1.77 | 0.97 | 0.42 | 0.44 | 0.04 | NS | -0.01 |
| wx2-4 | 4.00 | 3.11 | 1.22 | 0.62 | 0.68 | 0.08 | NS | 0.04 |
| wx4 | 5.00 | 1.66 | 0.83 | 0.44 | 0.40 | -0.11 | NS | -0.06 |
| SM13 | 5.00 | 2.20 | 0.98 | 0.44 | 0.55 | 0.19 | NS | 0.11 |
| Mean | 6.08±0.43 | 2.62±0.29 | 1.13±0.09 | 0.53±0.04 | 0.57±0.04 | 0.06±0.05 | — | 0.04±0.03 |

**Table S3** Diversity parameters for the Chinese fir offspring.

| Locus | Na | Ae | I | Ho | He | F | HW | F(Null) |
| --- | --- | --- | --- | --- | --- | --- | --- | --- |
| wx1 | 6.00 | 2.20 | 1.08 | 0.63 | 0.55 | -0.15 | ** | -0.09 |
| wx2 | 8.00 | 3.86 | 1.49 | 0.84 | 0.74 | -0.13 | * | -0.06 |
| wx2-3 | 6.00 | 1.71 | 0.78 | 0.44 | 0.42 | -0.05 | NS | -0.02 |
| wx2-6 | 11.00 | 3.48 | 1.56 | 0.78 | 0.71 | -0.09 | NS | -0.05 |
| wx6 | 5.00 | 2.06 | 0.92 | 0.55 | 0.52 | -0.07 | NS | -0.03 |
| wx7 | 7.00 | 2.16 | 1.13 | 0.55 | 0.54 | -0.03 | NS | -0.03 |
| wx2-11 | 5.00 | 2.38 | 0.99 | 0.64 | 0.58 | -0.10 | NS | -0.05 |
| wx2-8 | 9.00 | 3.11 | 1.40 | 0.68 | 0.68 | 0.00 | NS | 0.00 |
| wx8 | 7.00 | 2.04 | 1.12 | 0.57 | 0.51 | -0.12 | ** | -0.08 |
| wx2-4 | 4.00 | 3.00 | 1.14 | 0.78 | 0.67 | -0.17 | ** | -0.08 |
| wx4 | 7.00 | 1.70 | 0.86 | 0.45 | 0.41 | -0.09 | NS | -0.06 |
| SM13 | 5.00 | 2.37 | 1.01 | 0.48 | 0.58 | 0.18 | * | 0.09 |
| Mean | 6.67±0.57 | 2.51±0.20 | 1.12±0.07 | 0.61±0.04 | 0.57±0.03 | -0.07±0.03 | — | -0.04±0.01 |

**Table S4** Summary of the effective population size (Ne)analysis. Ne, Effective population size (Ne) calculated with LD method with 95% confidence intervals; ♂Ne, male effective population size; ♂Ne/N, the ratio of male effective population size to the census population size; ♂Ne (P), Ne calculated including the pollen contamination.

|  | Ne | CI-lower | CI-high | ♂Ne | ♂Ne/N | ♂Ne (P) |
| --- | --- | --- | --- | --- | --- | --- |
| Parents | 49.00 | 34.50 | 75.00 | — | — | — |
| Offspring | 62.30 | 51.80 | 79.50 | 37.57 | 0.54 | 76.38 |

**Table S5** Variance analysis of the number of male and female flowers. Df: degree of freedom, MS: mean squares, F: F-statistic, P: *p*-value.

| Trait | Source of variation | Df | MS | F | P |
| --- | --- | --- | --- | --- | --- |
| Female flower | Parents | 68 | 2.084 | 3.489 | *p*<0.001 |
|  | Residuals | 183 | 0.597 |  |  |
| Male flower | Parents | 68 | 1.826 | 2.634 | *p*<0.001 |
|  | Residuals | 183 | 0.693 |  |  |
